# Posterior reversible encephalopathy syndrome with extensive cytotoxic edema after blood transfusion: a case report and literature review

**DOI:** 10.1186/s12883-018-1194-1

**Published:** 2018-11-12

**Authors:** Yoshitsugu Nakamura, Masakazu Sugino, Akihiro Tsukahara, Hiroko Nakazawa, Naomune Yamamoto, Shigeki Arawaka

**Affiliations:** 1 Division of Neurology, Aino Hospital, 11-18 Takadacho, Osaka, Ibaraki 567-0011 Japan; 2 Division of Internal Medicine, Aino Hospital, 11-18 Takadacho, Osaka, Ibaraki 567-0011 Japan; 3 Division of Neurology, Department of Internal Medicine IV, Osaka Medical College, 2-7 Daigakumachi, Takatsuki, Osaka 569-8686 Japan

**Keywords:** Posterior reversible encephalopathy syndrome, Blood transfusion, MRI, Cytotoxic edema, Neurological sequelae, Case report

## Abstract

**Background:**

Posterior reversible encephalopathy syndrome (PRES) is described as a clinical-radiological disease entity with good prognosis. In brain MRI, PRES generally presents with vasogenic edema. Although PRES is induced by various causes, a small number of PRES cases have occurred after red cell blood transfusion. It is unclear whether there are characteristic features in PRES after blood transfusion.

**Case presentation:**

Here, we report a case of 75-year-old Japanese woman who had acute exacerbation of subacute anemia by bleeding from gastric ulcer. After receiving a red cell blood transfusion, she showed disturbance of consciousness with extensive cytotoxic and small vasogenic edema in the occipitoparietal area on brain MRI. She was diagnosed as PRES and suffered irreversible impairments of visual acuity and fields in both eyes. We summarized and discussed clinical features of cases with PRES after blood transfusion.

**Conclusions:**

A total of 21 cases including the present one have been reported as PRES after blood transfusion. Of the cases, 20 of 21 were female, and 15 of 17 developed PRES in the course of chronic anemia lasting over 1 month. Anemia was severe in 15 of 20 cases, with hemoglobin levels < 3.5 g/dl. In 14 of 17 cases, hemoglobin levels increased to 5 g/dl by red cell blood transfusion until the onset of PRES. On brain MRI, 2 of 21 cases showed cytotoxic edema and 3 of 21 cases showed irreversible neurological disturbance. In this patient, the occurrence of PRES in subacute anemia and the presence of extensive cytotoxic brain edema with irreversible neurological deficits were characteristic points. When treating severe anemia, even with a subacute progression, we should consider a possibility that PRES occurs after blood transfusion with extensive cytotoxic brain edema and irreversible neurological changes.

## Background

Posterior reversible encephalopathy syndrome (PRES) was first described as a clinical and radiological disease entity by Hinchey and colleagues in 1996 [1]. Usually, PRES presents subcortical vasogenic brain edema in patients with acute neurological symptoms, such as conscious disturbance, headache, seizure, and various visual disturbances. Vasogenic edema was observed in the bilateral parieto-occipital regions as elevation of apparent diffusion coefficient (ADC) values in brain magnetic resonance imaging (MRI) [1, 2]. The prognosis of PRES is generally good in terms of improvement and reversal of radiological and clinical findings. However, a previous paper reported atypical cases. These showed cytotoxic edema, neurological sequelae, and lack of hypertension [2]. Cytotoxic edema was observed as hyperintensity on diffusion weighted images (DWI) and decrease of ADC values in brain MRI.

PRES occurs in association with severe hypertension, renal failure, eclampsia, autoimmune disorders, and exposure to immunosuppressive agents. The clinical features of PRES caused by preeclampsia-eclampsia or calcineurin inhibitors are shown to be different from those caused by other triggers [3–5]. PRES is seen after red cell blood transfusion in a small number of patients [6–21]. However, there was no study analyzing these cases of PRES after blood transfusion. The clinical features of PRES after blood transfusion remains unclear. Here, we report a patient who had PRES after being treated for severe anemia with red cell blood transfusion. This case showed cytotoxic edema in the occipitoparietal regions on brain MRI, and developed irreversible visual disturbance. These features were different from typical PRES. We review previous cases of PRES after red cell blood transfusion and discuss the features of these cases with the supposed mechanism of brain damages caused by an increase in the hemoglobin (Hb) concentration.

## Case presentation

A 75-year-old woman (height 132 cm, weight 34 kg) consulted a medical doctor with loss of appetite lasting for 1 week. She had taken aspirin, celecoxib, and amlodipine because of angina and hypertension. Laboratory examinations showed that her Hb decreased modestly to 10.7 g/dl. Two weeks later, she was admitted to our hospital because her anorexia had worsened day by day. On admission day 1, she was alert and fully oriented. Her blood pressure and pulse rate were stable. Laboratory examinations showed that the levels of Hb and hematocrit decreased remarkably to 4.8 g/dl and 15%, respectively. The values for white blood count and C-reactive protein (CRP) increased slightly to 9900 /mm^3^ and 4.64 mg/dl, respectively. Additionally, her albumin level decreased to 2.5 g/dl and the creatine level increased slightly to 1.14 mg/dl (estimated glomerular filtration rate was 41.7 ml/min). Chest and abdominal computed tomography (CT) failed to detect abnormal lesions. On admission day 2, she vomited large amounts of bright red blood. Her Hb level further decreased to 2.9 g/dl. She underwent an urgent transfusion of 560 ml of red blood cells. Endoscopic examination detected active bleeding from a gastric ulcer, and endoscopic clipping was performed against the bleeding lesion.

**Fig. 1:**
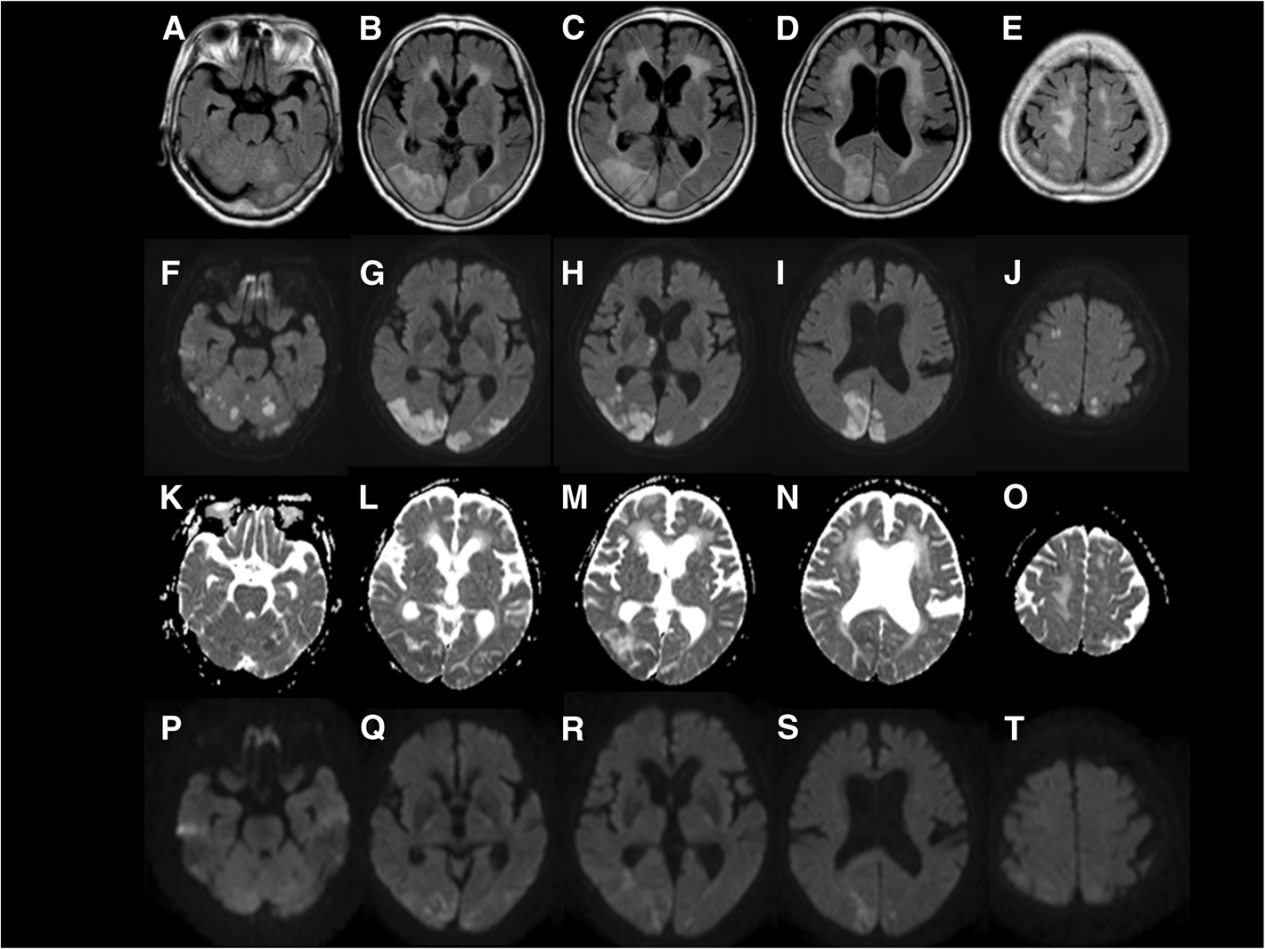
On admission day 2, axial fluid-attenuated inversion recovery (FLAIR) images show abnormal hyperintense areas bilaterally in the cerebellar hemispheres, watershed regions, and white and grey matter of the occipital and occipitoparietal lobes. Axial FLAIR images show hyperintense areas in white matter predominating in the periventricular region indicating leukoaraiosis (**a, b, c, d, e**). Axial diffusion weighted image (DWI) images show hyperintense areas in these lesions and the right thalamus (**f, g, h, i, j**). Apparent diffusion coefficient (ADC) map shows low signal intensities in the bilateral cerebellar hemispheres and watershed regions (**k, o**). ADC map shows low signal intensities in the cortical and subcortical regions with small areas of high signal intensity in the surrounding area (**l, m, n**). These image patterns indicate that the lesions were damaged by extensive cytotoxic edema with restricted vasogenic edema. Most hyperintense areas on DWI on 2 days after admission disappeared by 7 days after admission (**p, q, r, s, t**)

The next day, her Hb improved to 8.9 g/dl. However, she did not respond to verbal or pain stimuli despite her eyes having opened. She showed normal light reflexes without anisocoria. On brain MRI (fluid-attenuated inversion recovery, FLAIR), high signal intensities were seen in the bilateral cerebellar hemispheres, bilateral watershed regions, right thalamus, and white and gray matter of the bilateral occipital and occipitoparietal lobes. DWI also showed high signal intensities in these lesions, while ADC maps show low signal intensities in the cortical and subcortical regions with small high signal intensities in the surrounding area. These image patterns indicated that the lesions were damaged by extensive cytotoxic edema with restricted vasogenic edema (Fig. 1). Magnetic resonance angiography (MRA) showed no stenosis or occlusion of brain arteries. MR venography and 3-dimensional CT angiography was negative for sinus thrombosis, although the left transverse sinus was hypoplastic.

During this process, the maximum systolic blood pressure was 130 mmHg. Laboratory examinations showed no abnormality in liver and coagulatory functions. The level of serum creatinine was stable. Autoantibodies, such as anti-nuclear antibody, anti-thyroid antibody, and anti-neutrophil cytoplasmic antibody, were negative. There was no mutation at position 3243 of the mitochondrial DNA. The cerebrospinal fluid was clear, colorless and acellular with a total protein level of 41 mg/dl and a glucose level of 79 mg/dl. Transthoracic echocardiography showed that myocardial wall motion was normal without foramen ovale or left ventricular thrombus. Holter electrocardiogram showed no atrial fibrillation. From these examination results, the clinical course, and characteristic MRI findings, we considered the possibility of PRES and performed conservative treatment by monitoring the fluctuations of blood pressure and vital signs.

**Fig. 2:**
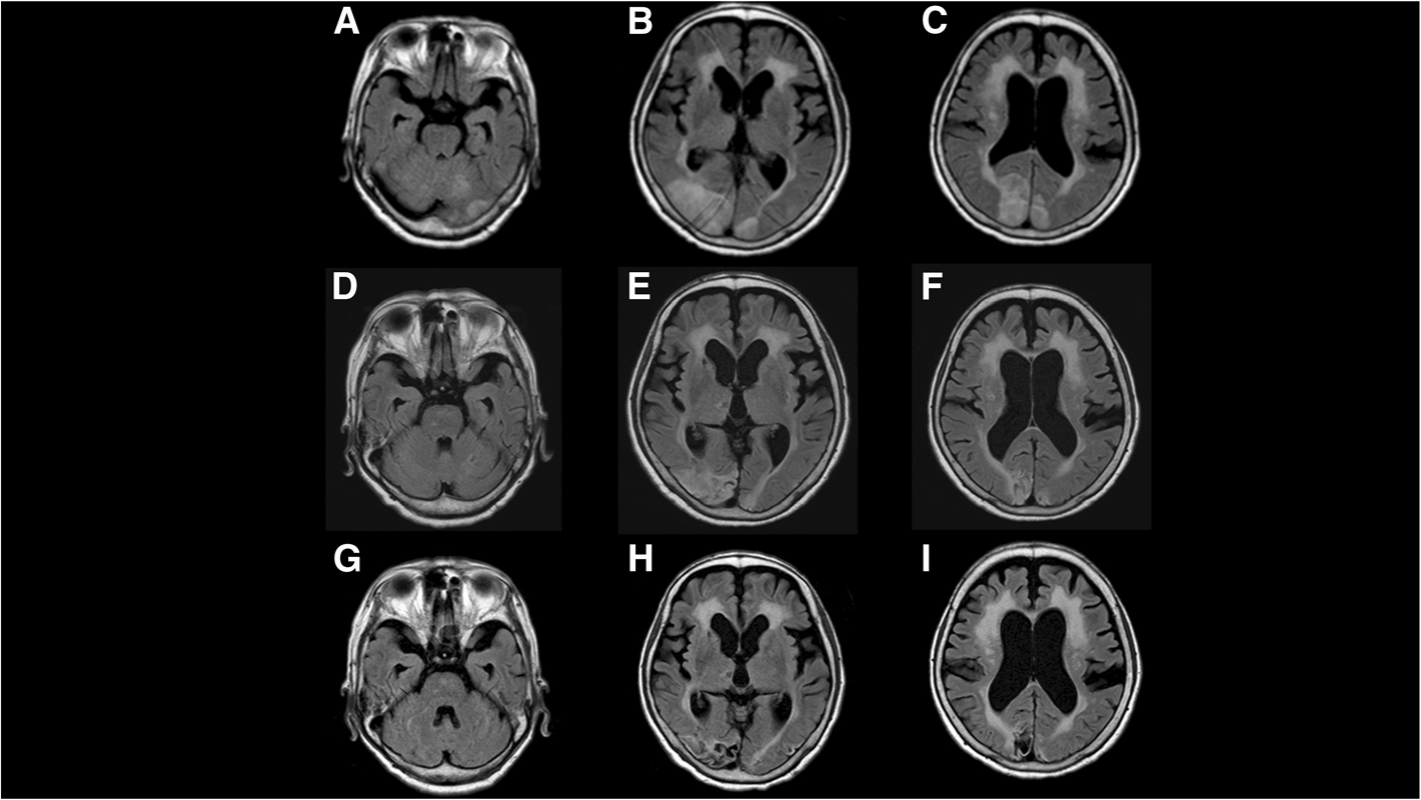
Hyperintense areas on axial fluid-attenuated inversion recovery (FLAIR) image at 2 days after admission (**a, b, c**) gradually disappear at 2 months after admission (**d, e, f**) and 4 months after admission (**g, h, i**). However, at 4 months after admission several cystic changes remained in the cerebellar hemisphere, occipital region, and right thalamus (**g, h, i**)

On admission day 7, her responsiveness to external stimulation improved. However, her visual acuity had declined to 20/100 in the right eye and 20/200 in the left eye, and visual field test showed defects of the left lower quadrants in both eyes. Her optic disc and retina were normal. Two months later, her Mini-Mental State Examination score improved to 16 from 8 on admission day 14. However, the disturbances of visual acuity and fields remained unrecovered. When compared with MRI findings, high signal intensities on DWI images were diminished on admission day 7 (Fig. 1). In addition, high signal intensities on FLAIR images were diminished until 4 months after onset, and these lesions alternatively involved low signal intensities, indicating cystic change (Fig. 2). There were no findings showing cardiogenic embolism and cerebral venous thrombus. The cystic lesion in posterior lobe seemed to represent ischemic change, because PRES is reported to cause ischemic change as a complication [22].

## Discussion and conclusions

### Methods

This review was based on a literature search for articles concerning PRES after blood transfusion that were published from 1997 through August 2017. By using the Medline database (PubMed, National Library of Medicine, Bethesda, MD; keywords: posterior reversible encephalopathy syndrome and blood transfusion) and further checking the reference lists of articles, 20 cases of PRES after red cell blood transfusion were identified. In this report, a total of 21 cases including the present one, were reviewed.

The distribution of brain lesions was divided into three areas: anterior circulation (AC), posterior circulation (PC), and deep structure (DS) territories. AC includes the frontal, temporal and parietal lobes. PC includes the occipital lobe, cerebellum and brainstem. DS includes the basal ganglia, deep white matter and corpus callosum [23]. Patients with sickle cell anemia who developed PRES after blood transfusion were excluded because sickle cell anemia itself was reported to cause PRES [24].

### Results of literature review

Table 1 shows the characteristics of 21 cases of PRES after blood transfusion. The mean age of onset was 43.6 years (range 6–77 years). Twenty cases (95%) were in females, and all adult cases were in females. The primary causes of anemia varied, such as gynecological disease (10 cases), renal failure (3 cases), iron deficiency anemia (2 cases), aplastic anemia (2 cases), cancer surgery (2 cases), thalassemia (1 case), and gastrointestinal bleeding (1 case). In the majority of PRES cases after blood transfusion (15 (88%) of 17 assessable cases) had been affected by chronic anemia lasting over 1 month. The mean Hb value before blood transfusion was 3.4 g/dl (range 1.4–9.2). Severe anemia (Hb < 3.5 g/dl) was observed in 15 (75%) of 20 assessable cases. The mean Hb value after blood transfusion was 6.7 g/dl (range 3.2–10.4). Hb increased to ≥5 g/dl in 14 (82%) of 17 assessable cases just before the onset of PRES. The mean onset of neurological symptoms after blood transfusion was 7.1 days (range 1–18 days). The period from blood transfusion to the occurrence of PRES is known to depend on some factors, including the patient’s condition, disease complication, and total amount and speed of blood transfusion [19]. Neurological symptoms were encephalopathy (9 cases, 43%), seizure (15 cases, 71%), headache (13 cases, 62%), visual disturbance (11 cases, 52%), and focal deficit (2 cases, 10%). The number of cases with hypertension was small (8 cases, 38%), in contrast to the data showing that 80–85% cases of PRES exhibit hypertension [2]. Neurological sequelae, such as consciousness disturbance, visual disturbance and hemiplegia, were found in 3 cases (14%). PRES brain lesions after blood transfusion were seen in the PC territory (20 cases, 95%), the AC territory (13 cases, 62%), and the DS territory (3 cases, 14%). On brain MRI, 2 cases (9%) presented with cytotoxic edema, and vasoconstriction was identified in 9 cases (43%). Vasoconstriction was caused by cerebral angiography in 3 cases and MR angiography in 6 cases.

**Table 1:** Previous reports PRES after blood transfusion and our case

Patient No.	Age/Sex	Cause of anemia	Course of anemia	Hb(g/dl) pre/post BT	Volume of BT (ml)	Symptom onset after BT (days)	Clinical finding	Lesion distribution	Cytotoxic edema	Vasoconstriction	Hypertension	Sequelae	Reference
1	45/F	Myoma uteri	Chronic	2.0/10.0	800	2	S, H	AC, PC	No	Yes	Yes	None	6
2	48/F	Myoma uteri	Chronic	3.0/8.0	1000	6	E, S, F	AC, DS	No	Yes	No	None	7
3	47/F	Aplastic anemia	Chronic	1.5/10.9	NR	7	E, S, H, V	PC	No	Yes	No	None	8
4	58/F	Cancer surgery	NR	7.7/10.9	1400	9	E, S	PC	No	No	Yes	E	9
5	77/F	Cancer surgery	Acute	9.2/13.3	2800	18	E, S	PC	No	No	Yes	None	9
6	32/F	Myoma uteri	Chronic	5.7/12.5	1600	5	H	PC	No	Yes	No	None	10
7	11/M	Iron deficiency anemia	NR	NR/NR	NR	NR	S, V	AC, PC	No	No	No	None	11
8	42/F	Renal failure	Chronic	5.7/11.7	400	6	S, H, V	PC	No	Yes	No	None	12
9	56/F	Corpus uteri cancer	Chronic	2.0/9.2	2000	6	E, S, V	PC	No	No	Yes	None	13
10	28/F	Aplastic anemia	Chronic	3.2/9.6	1640	8	H, V	AC, PC	No	No	No	None	14
11	57/F	Iron deficiency anemia	Chronic	2.0/10.0	1120	10	S, H	AC, PC	No	No	Yes	None	14
12	50/F	Hypermenorrhea	Chronic	1.5/NR	3000	NR	S, H	AC, PC	No	Yes	Yes	None	15
13	46/F	Myoma uteri	Chronic	1.4/NR	2500	15	H, V	AC, PC	Yes	Yes	Yes	None	15
14	36/F	Hypermenorrhea	Chronic	1.4/11.3	1120	12	E, S	AC, PC, DS	No	No	No	V, F	16
15	6/F	Thalassemia	NR	4.8/NR	280	2	E, H	PC	No	No	No	None	17
16	36/F	Myoma uteri	Chronic	1.7/8.8	560	2	E, S, H, V	AC, PC	No	No	Yes	None	18
17	45/F	Renal failure	Chronic	3.4/7.9	800	4	H, V	AC, PC	No	Yes	No	None	19
18	47/F	Renal failure	Chronic	3.0/10.4	750	NR	S, H, V	PC	No	Yes	No	None	19
19	40/F	Hypermenorrhea	Chronic	3.1/8.6	840	4	S, H, V, F	AC, PC	No	No	No	None	20
20	35/F	Abortion	NR	3.4/13.8	700	10	S	AC, PC	No	No	No	None	21
21	75/F	Gastrointesional bleeding	Subacute	2.9/8.9	560	1	E, V	AC, PC, DS	Yes	No	No	V	Our case

*Abbreviations*: *PRES* posterior reversible encephalopathy syndrome, *NR* not reported, *Hb* hemoglobin, *BT* blood transfusion, *E* encephalopathy, *S* seizure, *H* headache, *V* visual disturbance, *F* focal deficit, *AC* anterior circulation, *PC* posterior circulation, *DS* deep structure

## Discussion

Although the exact mechanism of PRES after blood transfusion is unclear, a rapid increase in the Hb level and viscosity by the blood transfusion is thought to trigger the occurrence of PRES. This increase could induce acute vascular endothelium dysfunction and an elevation of vascular resistance, leading to endothelial damage and extravascular leakage of fluid and macromolecule in the brain. Also, the velocity of brain blood flow is shown to increase in patients with severe anemia [25]. It raises an idea that rapid elevation of vascular resistance or vascular constriction factors in blood products damages vascular endothelial cells [26]. Consequently, these changes are thought to cause PRES [6]. However, a previous paper showed that anemia itself caused PRES under a hemorrhagic shock state with sepsis or multiple organ failure [27]. The present patient was not consistent with that prior case because our patient had no signs or symptoms of sepsis or multiple organ failure. There was a possibility that rapid elevation of Hb levels affected the occurrence of PRES. The elevation of Hb levels by blood transfusion is dependent on the volume of circulating blood, which is associated with the body weight [28]. It is possible that Hb levels rapidly elevated from 2.9 g/dl to 8.9 g/dl by transfusing 560 ml of blood, because the body weight of this patient was low. This rapid elevation of Hb levels may affect the occurrence of PRES.

There are two characteristic points distinguishing the present patient. First, the patient presented with extensive cytotoxic edema on brain MRI. In cases with PRES after blood transfusion, the frequency of cytotoxic edema was less than that of vasogenic edema. Cytotoxic edema was found in only 11–30% of previous cases with PRES [2, 29]. However, it is unclear how cytotoxic edema occurs in PRES after blood transfusion, and whether the cytotoxic edema in PRES causes irreversible damages is under debate [2, 4, 22, 29–33]. In addition, the present patient showed cytotoxic edema over an extensive area as compared with other cases with cytotoxic edema. In the present patient, this extensive cytotoxic edema may have helped to cause the irreversible visual disturbance. Indeed, while the number of cases with extensive cytotoxic edema is very small, those cases are generally associated with irreversible changes and incomplete clinical recovery [2, 22, 29–32]. Therefore, the clinical course of the present patient may suggest that rapidly correcting anemia with red cell blood transfusion should be avoided to prevent PRES. It supports an idea that cytotoxic edema causes irreversible damages in PRES. To address this issue, it is necessary to collect similar cases with neurological sequelae.

Second, the period of anemia in the present patient was shorter those that reported in previous cases. Most cases (88% of all reported cases of PRES after blood transfusion) had had chronic anemia lasting over 1 month [6–8, 10, 12–16, 18–20]. In these typical cases, it assumed that a rapid improvement of oxygenation by blood transfusion induces PRES by disturbing the balance of vessels, which is maintained by chronic hypoxic vasodilation [6]. In the present patient, the period of anemia during which Hb decreased from 10.7 to 2.9 was 14 days. The presence of other factors may exacerbate the occurrence of PRES. Severe anemia itself (Hb 2.9 g/dl) may affect the occurrence of PRES. Also, previous reports have demonstrated that elevation of CRP levels [34], hypoalbuminemia [35, 36], and renal injury [37] are candidate factors affecting the occurrence of PRES. Elevation of CRP levels has been shown to exacerbate PRES by increasing the vulnerability of the blood–brain barrier by inflammation-associated endothelial damage [34]. Hypoalbuminemia affects the development of edema in PRES by reducing colloid osmotic pressure [35, 36]. Renal injury is reported to mediate endothelial cell damage, which is a poor prognosis factor [37]. The present patient presented with elevated CRP levels, hypoalbuminemia, and renal injury. These factors may affect the occurrence of PRES after blood transfusion in patients with subacute severe anemia.

Finally, most cases of PRES after blood transfusion are female. The proportion of female PRES patients after blood transfusion is much greater than the proportion of female patients among all PRES patients, including eclampsia, because previous reports show that female patients account for 56–68% of all PRES patients [22, 29, 33, 34, 36–40]. The reason why many cases with PRES after blood transfusion were female remained unclear. About half of PRES after blood transfusion accompanied vasoconstriction. In general, the frequency of vasoconstriction is reported to be 18% in PRES [22, 37]. This suggests that the frequency of vasoconstriction in PRES after blood transfusion is higher than that in PRES induced by other causes. Decrease in estrogen levels may affect to cause vasoconstriction [41, 42], because estrogen suppresses vascular constriction [43]. Also, there is a tendency that almost papers concerning PRES after blood transfusion were published from Asia [6, 8–17, 19, 21]. To clarify whether genetic factors associate with the occurrence of PRES after blood transfusion, it is necessary to further analyze clinical features by accumulating cases with PRES after blood transfusion.

## Conclusion

In general, PRES after blood transfusion occurs in patients with chronic severe anemia and it appears vasogenic edema in the brain with good prognosis. However, as shown in this patient, PRES after blood transfusion rarely occurs in patients with subacute severe anemia and it caused irreversible damages. Clinical findings of this patient suggest that irreversible damages may be caused by extensive development of cytotoxic edema in the brain. Additionally, if the anemia is urgently corrected by blood transfusion, clinicians should check for the presence of risk factors, including elevation of CRP levels, hypoalbuminemia, renal injury, and female sex, and perform the blood transfusion slowly with carefully monitoring the increase of Hb levels.

## Abbreviations

AC: Anterior circulation
ADC: Apparent diffusion coefficient
CRP: C-reactive protein
CT: Computed tomography
DNA: Deoxyribonucleic acid
DS: Deep structure
DWI: Diffusion weighted image
FLAIR: Fluid attenuated inversion recovery
Hb: Hemoglobin
MRA: Magnetic resonance angiography
MRI: Magnetic resonance imaging
PC: Posterior circulation
PRES: Posterior reversible encephalopathy syndrome
